# Investigation of water sorption and aluminum releases from high viscosity and resin modified glass ionomer

**DOI:** 10.4317/jced.56381

**Published:** 2020-09-01

**Authors:** Numan Aydın, Serpil Karaoğlanoğlu, Elif Aybala-Oktay, Serdar Çetinkaya, Onur Erdem

**Affiliations:** 1 DDS, PhD. University of Health Sciences, Gulhane Faculty of Dentistry, Department of Restorative Dental Treatment, Ankara, Turkey; 2 PhD. University of Health Sciences, Gulhane Faculty of Pharmacy, Department of Toxicology, Ankara, Turkey

## Abstract

**Background:**

High viscosity glass ionomer cement (HVGIC) and resin-modified glass ionomer cement (RMGIC) have recently been clinically preferred thanks to their numerous advantages. However, initial moisture contamination has a negative effect on the mechanical and physical properties of these cements. The aim of this study was *in vitro* of HVGICs and RMGICs, with and without surface protection, on water sorption, solubility and release of aluminum.

**Material and Methods:**

In this study, as HVGICs; Equia Forte, IonoStar Plus, Riva Self Cure; as RMCIS, Ionolux and Riva Light Cure; and as control, Z250 universal composite was used. Equia coat, Voco varnish and Riva coat were chosen as surface protective. Water sorption and solubility levels of the samples were measured according to ISO 4049:2009. Al levels released from samples were determined by graphite furnace atomic absorption spectroscopy (GFAAS) for 7, 14 and 21 days. Statistical evaluation of the results was made using one-way variance analysis (ANOVA) and Tukey post-hoc test (*p*<0.05).

**Results:**

RMGICs from restorative materials showed more water absorption than HVGICs, but no differences in solubility. Among the materials tested, the water absorption values of the HVGIC and RMGIC materials without surface protection were higher than those with the surface protection (*p*<0.001).

**Conclusions:**

It was determined that the Al release of HVGIC and RMGIC groups with the surface protection were lower in all time periods than the groups without surface protection (*p*<0.001). The application of surface protection effectively reduced water sorption and Al release from HVGICs and RMGICs.

** Key words:**Highly viscous glass ionomer cement, resin coating, aluminum release, water sorption, solubility.

## Introduction

Glass ionomer cements were first introduced to dentistry by Wilson and Kent in 1972 (1). Subsequently, resin-modified glass ionomer cements (RMGIC) were introduced into the market. This restorative materials powder part is composed of fluoroaluminosilicate glass powders, and the liquid part is composed of HEMA (2-hydroxyethyl methacrylate), methacrylate groups, polyacrylic acid, tartaric acid and water (2,3).

In recent years, high viscosity glass ionomer cements (HVGIC) have been developed in order to improve the weak mechanical properties of conventional CGICs and wear resistance against occlusal forces in clinical applications and to extend the indication areas limited to class I and class V restorations (3). In addition, these materials are intended to be an alternative to composite resin and amalgam as the material in permanent restorations of the teeth. Wear resistance, surface hardness, bending and compression resistance of these cements, whose hardening mechanisms are similar to CGICs, have been increased. The fluorides and biocompatibility of these cements are similar to CGICs (4). Water sorption, which affects the physical, chemical and mechanical properties of all dental restorative materials, is one of the factors that affect the clinical success of dental restorative materials and cannot be kept under control completely. The hypersensitivity of CGICs to moisture is also evident in HVGIC and RMGIC. Due to the hydrolysis of the cement matrix, water sorption leads to the degradation of cements in time and leads to the loss of surface properties, edge integrity, aesthetic appearance and consequently increase of the deteriorations in restorations (5,6). In order to eliminate these disadvantages, it is recommended to provide a protection with a surface covering application for periods ranging from 1 hour to 2 weeks, in order to protect from moisture interaction after dental restorations are made (7,8). With the developments in surface covering systems, light cured surface coverings have emerged as optimal surface protection agents. In recent years, nano-filler surface coverings combined with HVGICs have been introduced to the market.

The biocompatibility of CGICs are significantly influenced by the release of fluorine (F) and aluminum (Al) from their structure. Al is an element which is taken into the organism from different sources and has potentially toxic effects (9). There are studies about the association of Al with the pathogenesis of Alzheimer’s disease. Furthermore, it has been reported to play a role in other neurological diseases including Parkinson, pathogenesis of amyotrophic lateral sclerosis, skeletal and hematological diseases (10,11). After restoration, fluoride release of the glass ionomer cements used in restorative dentistry into the mouth, as well as other elements such as aluminum, calcium (Ca) and sodium (Na) are available in literature (12,13). In the studies, it was found that the Al ions on the cement surface were released at most at the very first day during the polymerization of CGICs, and the rest were trapped in the depths of the matrix (13). In the researes, the amount of fluoride released from CGIS was investigated. However, water sorption, dissolution and release of Al were neglected when these materials were used with and without surface protection. The aim of this study was *in vitro* examination of the effect of usage of HVGICs and RMGICs, with and without surface protection, on water sorption, solubility and release of aluminum.

## Material and Methods

In our study as HVGIC; Equia Forte (GC Corporation, Japan), IonoStar Plus (VOCO GmbH, Germany), Riva Self Cure (SDI, Australia) and as RMGIC; Ionolux (VOCO GmbH, Germany) and Riva Light Cure (SDI, Australia) was used (Table 1). Each material’s same brand protector was selected as the surface protector (Equia coat, Voco varnish and Riva coat). The Filtek Z250 (3M ESPE, USA) universal composite was used as a control (Table 1).

**Table 1:**
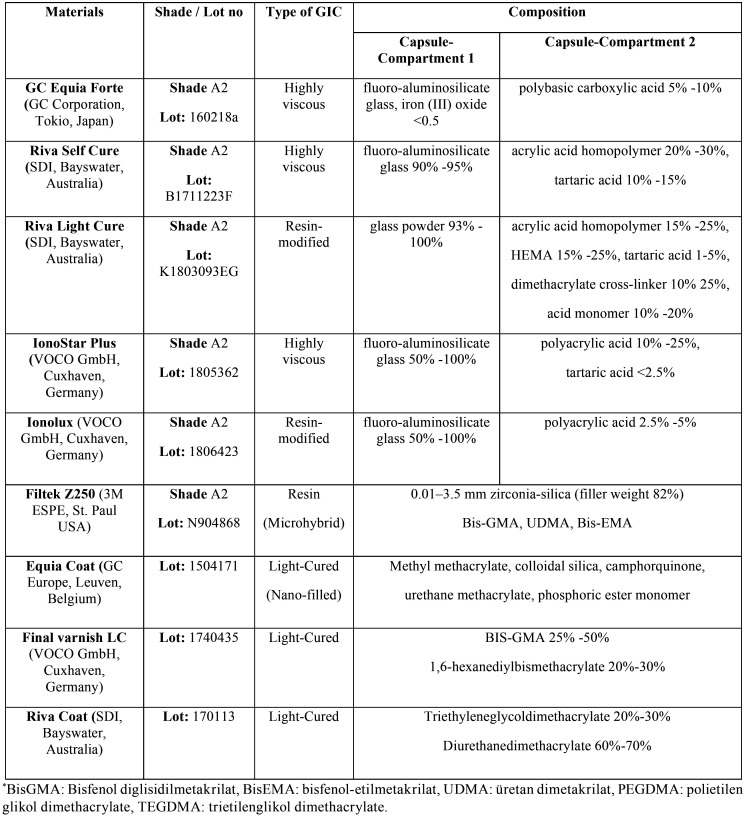
HVGICs, RMGICs and surface protectors used in the study.

-Preparation of samples 

Each of the materials examined in this study was prepared using 10 sample (15x1mm) silicone modes as specified in ISO 4049:2009 (14), for polymerization of HVGICs, it was polymerized as long as the polymerization period suggested by the manufacturer. For polymerization of RMCIS and composite material, they were polymerized for the period suggested by the manufacturer (20 sec.), using LED light source (DTE LUX E, Germany, 1200 mw/cm2). The prepared samples were randomly divided into two groups (n:5) with and without surface protection. Each material’s same brand surface protection material was chosen as surface protection material. They were polymerized with the same led light source in accordance with the recommendations of the manufacturer (10 sec.).

-Water sorption and solubility test

 Water sorption and solubility levels of the samples were performed as specified in ISO 4049:2009 (14). Prepared samples are placed in desiccator containing anhydrous calcium chloride and kept for 22 hours at (37 ± 1) oC, then at a similar desiccator for 2 hours at (23 ± 2) oC. The weight of each sample taken from the desiccator was measured (within 15 sec.) with precision electric scales (Mettler AT201, Switzerland). This was continued until a constant weight value was obtained (0.1 mg) and the measured values were recorded. (M1)

Samples were kept in 10 mL of ultrapure water (Veolia, UK) in incubator (Heraeus D- 6450 Hanau, California) at 37+1 oC for 7 days after the measurement and then removed from the water. Their surfaces were dehumidified with the blotting paper and then re-weighed. (M2) The weighed samples were placed in the desiccator with anhydrous calcium chloride and was kept for 22 hours at (37 ± 1) oC, then kept at the desiccator for 2 hours at (23 ± 2) oC. The weight of each sample taken from the desiccator was measured (within 15 sec.) with precision electric scales (Mettler AT201, Switzerland). (M3)

In accordance with the ISO 4049:2009 (14), values for the water sorption (Wsp) and the solubility (Wsl) at specific times were calculated using the following equations, respectively.

Wsp = [M2 – M3] ÷ V and Wsl = [M1 – M3] ÷ V 

M1 = is the conditioned mass, in micrograms, prior to immersion in water (μg)

M2 = is the mass of the specimen, in micrograms, after immersion in water for 7 days (μg)

M3 = is the mass of the reconditioned specimen, in micrograms (μg) 

V = is the volume of the specimen, in cubic millimetres (mm3)

-Determination of Al levels

After measurement of the first dry weight of samples (M1, the samples were incubated in the incubator (Heraeus D-6450 Hanau, California) during the 21-day testing period in 10 mL of ultrapure water (Veolia, UK) at 37 ± 1 oC. The samples were collected for the first 7 days and then on the 14th and 21st days and the samples were transferred to 10 mL of propylene tubes. Water samples were kept at 4oC until the analysis time. Fresh ultrapure water was added in 10 mL volume to replace the water samples taken. This was done on days 7, 14 and 21. The values of Al in the ultrapure water were measured at the beginning and after each measurement process.

Al levels released from the samples were determined using Graphite Furnace Atomic Absorption Spectrometry (GFAAS) (Perkin Elmer Analyst 800, USA). Argon gas was used as the carrier gas in the analyzes. 1 g/L standard Al solution (Merck) was used for calibration. A 50 ng/mL stock solution was prepared from this solution with 0.2% HNO3 (Merck). The prepared solution was arranged to correspond to the concentration of 10, 20, 30, 40, 50 ng/mL for use in the calibration process by system software during the analysis. Calibration graph was prepared with the help of absorbance values corresponding to the concentration.

-Statistical Analysis 

Statistical data analysis was performed using SPSS 22.0 Statistical Program (SPSS Inc., Chicago, USA). The water sorption and solubility and Al release of the HVGICs and RMGICs used in the study were evaluated by using one-way variance analysis (ANOVA) and Tukey post-hoc test. *P*<0.05 was considered to indicate statistical significance.

## Results

Average water sorption values and standard deviations obtained as a result of our study are shown in Table 2-3. Statistically significant differences were found between the water sorption values of HVGIC and RMGIC (*p*<0.001). In addition, it was determined that all groups showed more water sorption than the control group (*p*<0.05). Maximum water sorption was detected in RMCIS groups at Riva Light Cure (11.76 μg/mm3) and Ionolux (11.35 μg/mm3), while the lowest water sorption value (9.66 μg/mm3) detected in Equia Forte, which is HVGIC (Table 2).

**Table 2:**
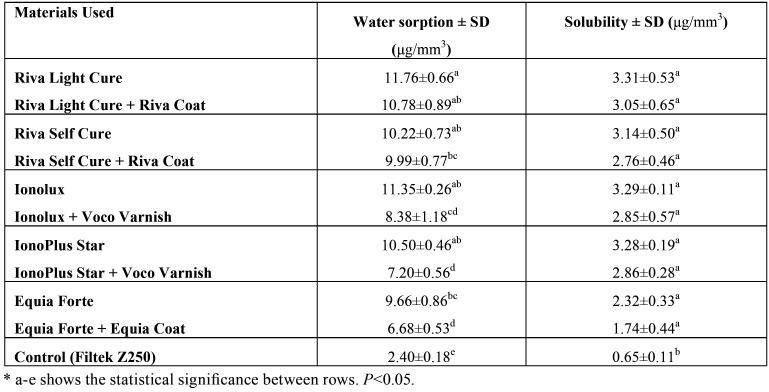
Effect of HVGIC and RMGIC surface protection application on water sorption and solubility.

**Figure 1:**
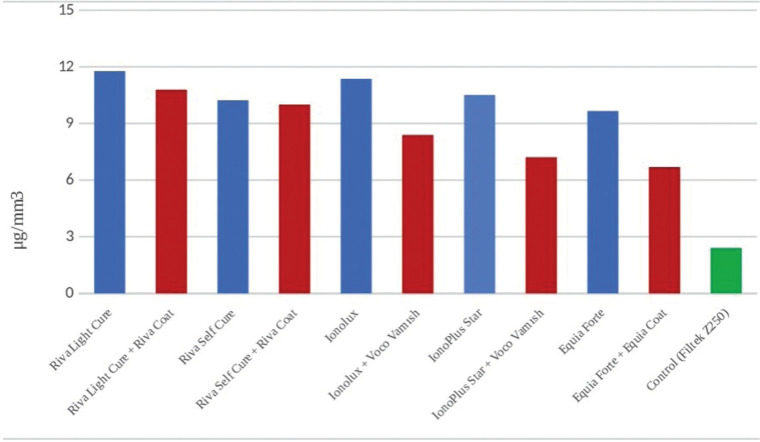
Water sorption of HVGIC and RMGIC.

HVGIC and RMGIC groups treated with surface protectors showed less water sorption than the non-protective group and more water absorption than the control group (*p*<0.05). Riva Light Cure + Riva Coat, which is RMGIC, has shown the highest water absorption value (10.78 μg/mm3), whereas Equia Forte + Equia Coat, which is HVGIC, has shown the lowest (6.86 μg/mm3) water sorption value (Table 2, Fig. 1).

When HVGIC and RMGIC groups were evaluated in terms of water sorption, there were no statistically significant difference between the Riva Light Cure and Riva Self Cure groups that have been applied with surface protection (*p*>0.05). However, statistically significant differences (*p*<0.05) were found between the Ionolux, IonoPlus Star, and Equia Forte groups that had been applied with surface protection (Table 2).

When HVGIC and RMGIC solubility values were examined, there was no statistically significant difference, although RMGICs had shown more solubility than HVGIC(p>0.05). There was no statistically significant difference when the groups of these cements with surface protection and without surface protection were compared (*p*>0.05). A statistically significant difference (*p*<0.001) was observed between the control group and all the other groups with and without surface protection (Table 2).

**Figure 2:**
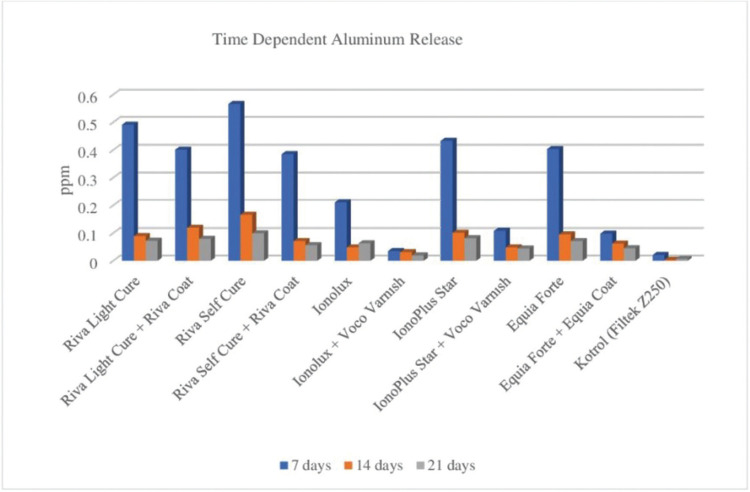
Time dependent Al release of HVGIC and RMGIC.

When the Al releases of restorative materials of HVGIC and RMGIC were evaluated according to weeks, the difference was statistically significant (*p*<0.001). At the end of the three weeks, the most Al release was detected with Riva Self Cure 0.831 ppm (parts per million), which is HVGIC, while the least Al release was detected Ionolux 0.089 ppm, which is a surface protection applied RMGIC. Besides, it was determined that groups of Riva Light Cure and Riva Self-Cure, Ionolux, IonoPlus Star and Equia Forte, treated with surface protection, releases less Al in all time periods than the groups without a surface protection (*p*<0.001). There was a statistically significant difference (*p*<0.001) between the control group and the groups with and without surface protection. It was observed that the maximum Al release was at the first week and decreased as the time period progressed ( Table 3, Fig. 2).

**Table 3:**
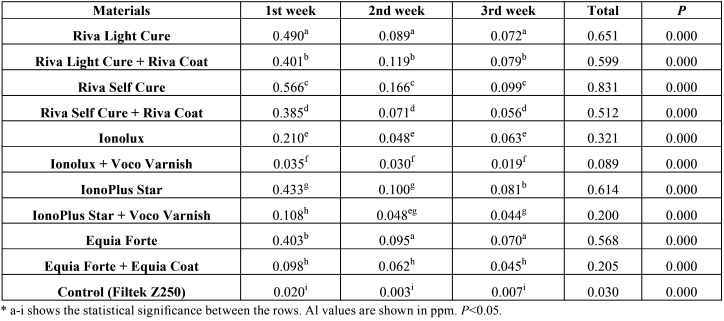
Time dependent Al release from HVGIC and RMGIC.

## Discussion

HVGICs and RMGICs were produced to overcome the inadequacies of aesthetic appearance of conventional glass ionomer cements, their sensitivity to water and weak mechanical properties and to benefit the clinical advantages of these cements. CGICs are clinically preferred because of their chemical bonding to tooth structures, fluorine release and reminelization (3,15). In spite of many advantages, initial moisture contamination can lead to a reduction in the mechanical and physical properties, deterioration of the matrix connection, coloration and edge breakage (16). The process of water absorption is, at present, explained by two theories. One theory hypothesizes that water molecules diffuse into microvoids where they interact with the resin matrix. The second theory proposes that water molecules bond to hydrophilic groups of the cement, resulting in hygroscopic expansion and increase of weight (17).

 In their study on the water absorption and solubility of glass ionomer cements, Lima *et al*. (18) found that RMGIC (Vitramer), the product of the same brand, showed more water absorption and solubility than CGIC (Ketac Molar Easymix). Cefaly *et al.* (19) reported in their studies that RMGIC cements showed more water absorption than composites. Studies in literature have attributed RMGICs’ having higher water absorption than that of CGIC (19) to the hydrophilicity of 2-hydroxyethylmethacrylate (HEMA) in the structure of RMGICs (20). Beriat and Nalbant (21) reported in their studies on the water absorption of RMGICs and HEMA release that there was a positive correlation between water absorption and HEMA release. In our study, RMGICs without surface protection (Riva Light Cure and Ionolux) were found to exhibit the highest water absorption and solubility.

Various surface preservatives such as light-cured bonding agent or varnish have been recommended to overcome the initial problems related to water absorption and dehydration in CGICs. After these recommendations, light-curing resin preservatives started to be applied on restorations (22) and in recent years HVGICs with capsule form and nano-filled resin coatings were made available to surgeons. In *in vitro* studies, it is stated that the application of surface protection to CGICs positively affects the polymerization process by maintaining water balance, control the hygroscopic expansion and increases its durability (22-24). In our study, nano-filled surface protector application on HVGIC and RMGIC was found to reduce water sorption, while HVGIC Equia Forte + Equia Coat showed the lowest water sorption (6.68 μg/mm3) values. While surface protectors Voco Varnish and Equia Coat reduces water sorption of HVGICs and RMGICs (*p*<0.05), there was no statistically significant difference in the sorption of water in Riva Coat applied groups (Riva Light Cure, Riva Self Cure) (*p*>0.05). In addition, it was found that surface protection application did not make a statistically significant difference while decreasing the solubility of HVGICs and RMGICs (*p*<0.05).

Surface protection application affects the water sorption of dental restorative material as well as other physical properties. Karaoglanoglu *et al.* (25) in their studies on the effects of surface protection application on dye sorption on glass ionomer, resin modified ionomer and polyacid modified composite resins, they stated that the use of surface protection significantly reduced the sorption of dye. Kanik *et al.* (26) stated that application of surface protective resin (EQUIA Coat) on HVGICs (EQUIA Fil and Riva Self Cure) increased the wear resistance of the material. Bonifacio *et al.* (27) also found that coating CIS (Fuji IX GP Extra and Ketac Molar) with resin (G coat) increased the wear resistance and bending strength of the material.

Al, which is the main component of HVGICs and RMGICs, plays an important role in acid-base reaction during the polymerization of cement and Al in the superficial layer is released (28). Studies have shown that Al accumulates in human body tissues such as brain, bone, liver and kidney (29,30). It has been shown that Al which is accumulated in bone tissue retards the formation and growth of hydroxyapatite crystals and may impair the process of mineralization (31). These days, it is widely accepted as a neurotoxicant now and may induce its toxic manifestations by exacerbating oxidative stress in brain. Moreover, mitochondria associated dysfunctions may also be regarded as causative factor for mediating Al toxicity (10). Various studies have indicated neuropathological, neurobehavioral, neurophysical and neurochemical changes following Al exposure (32,33).

Gjorgievska *et al.* (34) has shown in their studies that Al released by glass-ionomer and RMGIC fillings is absorbed by the tooth. Various types of tooth were restored by commercial glass-ionomer restorative materials, and then the release of aluminum and fluoride into artificial saliva was determined. Results showed that the lowest levels of Al were found in solutions where immature permanent teeth were stored than deciduous teeth, from which it was concluded that the immature permanent teeth have a higher affinity for aluminum than deciduous teeth (34).

Czarnecka *et al.* (35) in their study on ion release of glass ionomer cements, stated that the release of Al could be related to fluorine complexes, and found that aluminum, phosphorus and fluoride ions were released at all-time intervals and this release had a tendency to decrease over time. They also stated that the maximum release of Al was in the first week (41.2 ppm) and in the 4th week, the release of Al was reduced to a level (1.17 ppm) to be neglected. In another study by Czarnecka *et al.* (36) they have found that Ketac Endo (3.70 ppm) do more Al release than the Ketac Molar (1.73 ppm) and Fuji IX (1.71 ppm) in the analysis of glass ionomer cements in distilled water at the 1st week.

In the study of Okte *et al.* (37) on the fluorine and Al release of restorative materials using ion chromatography, the only observable aluminum release was from the conventional and resin-modified GICs during the first day in double-distilled water. In addition, the RMGIC Vitremer released a higher amount of aluminum than the conventional GIC Kavitan Plus. Savarino *et al.* (9) found that CGICs and RMGICs released more Al than the compomer in their study on the fluorine and Al release of compomer, conventional and resin-modified glass ionomer cements. They also reported that the ion release was highest during the initial polymerization (9). In our study in the distilled water at the end of the 1st, 2nd and 3rd weeks, the highest Al release was found in HVGIC Riva Self Cure (0.831 ppm) and the lowest in RMGIC Ionolux (0.321 ppm). However, Al release of HVGICs and RMGICs were found to be statistically higher than traditional composite (Filtek Z250) (*p*<0.001). In addition, similar to the results of other studies (9,35). Al release in all groups were found to be in a downward trend as the time period progressed.

When the studies in the literature are examined, there is no information about the effect of surface protective application on the release of Al. In our study, it was seen that the application of surface protector statistically significantly decreased (*p*<0.001) the Al release of HVGICs and RMGICs. In addition, the application of the surface protector decreased Al release at Ionolux (72.27%), Equia Forte (63.90%), IonoPlus Star (67.42%), Riva Light Cure (38.38%) and Riva Self Cure (7.98%). These results suggest that Voco Varnish and Equia Coat are more successful than Riva Coat on preventing Al release.

It is stated in the literature that glass ionomer cements can inhibit the polymerization reaction as a result of early water absorption (38). The application of effective (Voco Varnish and Equia Coat) surface preservatives on these materials in our study provides better polymerization by reducing water absorption. Enough polymerization of materials results in less Al release.

## Conclusions

As a result, materials used for restorative purposes are exposed to various factors such as stress, heat changes and chemical agents in the oral environment. As a result of these factors, water sorption, dissolution and release of Al occurs at HVGICs and RMGICs. The use of these materials, which are used extensively in restoration of deciduous and permanent teeth, not only reduces water sorption but also greatly reduces the release of Al, which is harmful to the human body. If the HVGICs and RMGICs are used with a nano surface protector with the correct indication, we think that the clinical use of the restorative material will be longer and will cause the patient the least damage.
